# Over two decades of research on the marine RNA virosphere

**DOI:** 10.1002/imt2.59

**Published:** 2022-10-17

**Authors:** Meng‐en Liao, Yunyi Xie, Mang Shi, Jie Cui

**Affiliations:** ^1^ CAS Key Laboratory of Molecular Virology & Immunology, Institut Pasteur of Shanghai, Center for Biosafety Mega‐Science Chinese Academy of Sciences Shanghai China; ^2^ University of Chinese Academy of Sciences Beijing China; ^3^ School of Medicine Sun Yat‐sen University Shenzhen Campus of Sun Yat‐sen University Shenzhen China; ^4^ Laboatory for Marine Biology and Biotechnology Pilot National Laboratory for Marine Science and Technology (Qingdao) Qingdao China

**Keywords:** ecology, marine RNA viruses, metagenomics, metatranscriptomics, taxonomy, virus evolution

## Abstract

RNA viruses (realm: Riboviria), including RNA phages and eukaryote‐infecting RNA viruses, are essential components of marine ecosystems. A large number of marine RNA viruses have been discovered in the last two decades because of the rapid development of next‐generation sequencing (NGS) technology. Indeed, the combination of NGS and state‐of‐the‐art meta‐omics methods (viromics, the study of all viruses in a specific environment) has led to a fundamental understanding of the taxonomy and genetic diversity of RNA viruses in the sea, suggesting the complex ecological roles played by RNA viruses in this complex ecosystem. Furthermore, comparisons of viromes in the context of highly variable marine niches reveal the biogeographic patterns and ecological impact of marine RNA viruses, whose role in global ecology is becoming increasingly clearer. In this review, we summarize the characteristics of the global marine RNA virosphere and outline the taxonomic hierarchy of RNA viruses with a specific focus on their ancient evolutionary history. We also review the development of methodology and the major progress resulting from its applications in RNA viromics. The aim of this review is not only to provide an in‐depth understanding of multifaceted aspects of marine RNA viruses, but to offer future perspectives on developing a better methodology for discovery, and exploring the evolutionary origin and major ecological significance of marine RNA virosphere.

## Highlights

- Advances in meta‐omics provide a surge of marine RNA virus information.
- Deep marine virus discovery contributes greatly to the megataxonomy of RNA viruses.
- Understanding diversity and biogeography helps reveal the significance of marine RNA viruses, including evolutionary trajectories, hierarchical taxonomy, and ecological importance.

## Graphical abstract

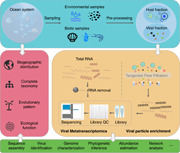

## INTRODUCTION

The first documented marine virus was discovered in a harbor crab (*Liocarcinus depurator*) in 1966 [1]; since then, numerous efforts have been made to explore viral pathogens in the ocean [2, 3, 4, 5]. Viruses were found to exist in high abundance in aquatic environments [6, 7], up to 2.5 × 10^8^ virions per milliliter, and to serve as an important component of the marine ecosystem through cell lysis and geochemical cycling [8, 9]. Despite extensive information on DNA viruses in natural environments, the features of RNA viruses in marine ecosystems, including their abundance, prevalence, distribution, ecology, and evolutionary patterns and trajectories, are poorly understood and summarized. Recently, studies to explore the global RNA virosphere [10] have shed light on their significance and function in natural niches [11, 12, 13, 14].

Covering over 70% of Earth's surface, marine habitats, with unique characteristics (e.g., hypoxia, limited light, hydrostatic pressure, and high inorganic chemical concentrations), provide a complex and changeable environment for aquatic organisms [15]. On the basis of depth, the open ocean is subdivided into five layers (i.e., epipelagic, mesopelagic, bathypelagic, abyssopelagic, and hadopelagic zone from the sea surface to the bottom), with significant differences in the environment due to unequal sunlight exposure [16]. Notably, similarity and heterogeneity of marine RNA virosphere between and within biogeochemically distinct layers have been revealed, and attempts have been made to predict how these relationships change along with environmental factors [17]. Notably, since the early 1990s, viruses have been found to impact the marine system, beyond as pathogens of marine cellular organisms, by affecting the host community and functioning as contributors to biogeochemical cycles. For example, the biological pump, transporting organic matter from the epipelagic layer to the deep zone, functions in global carbon cycling [18] in which viruses might play a vital role as they are estimated to kill numerous organisms per day [19]. Specifically, a recent study found a potential connection between marine carbon cycling and RNA viruses, since they probably infect marine hosts as a critical component of the biological pump and encode auxiliary metabolic genes (AMGs) involved in nutrient transport and photosynthesis. In addition, the abundance of some specific marine RNA viruses may strongly predict ocean carbon flux [20].

Recently, advanced high‐throughput sequencing technology has made it possible to explore the marine virome via a series of methods independent of culturing [21, 22]. In 2015, 5476 populations of marine viruses, most of which were phages, were reported [23] during a global scientific expedition, and this number was expanded to 15,222 in 2016 and 195,728 in 2019 [24, 25]. Several studies focusing on marine RNA viruses in recent years have been productive, and the amount of RNA virus information in public databases has increased sharply [26, 27, 28], bringing about challenges involving detecting divergent viruses and determining their taxonomy [29]. However, this dilemma is to some extent alleviated by deep phylogenetic analysis relying on RNA‐dependent RNA polymerase (RdRp), which has been used for sequence‐to‐sequence comparison and taxonomic assignment. And for viruses in the Kingdom of Orthornaviriae, multifaceted evidence like structure comparison and RdRp‐based hidden Markov model (HMM) can be used parallelly to address the problem of extreme sequence divergence of RdRp due to the ancient origin and high evolutionary change rates [10, 26, 30, 31, 32, 33].

**Figure 1:**
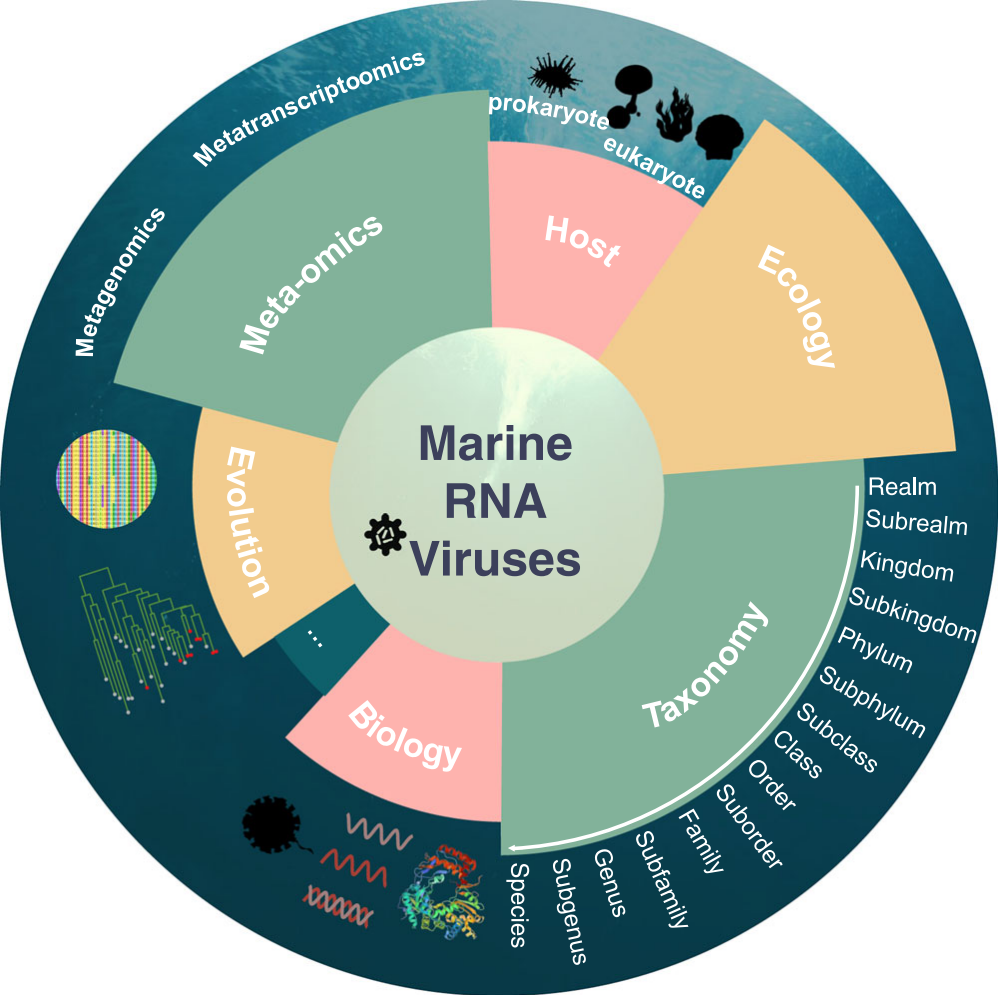
Various perspectives about marine RNA viruses. Many experimental efforts have already been made to characterize biological and epidemiological features and the host range of marine RNA viruses. Nowadays, a large number of marine RNA viruses have been discovered owing to the advent and advance of meta‐omics, including both metagenomics and metatranscriptomics, contributing to the hierarchical taxonomy and deep evolutionary process of RNA virosphere. Furthermore, with global sampling and comparative study, the ecological significance of marine RNA viruses is becoming clear. The taxonomy is generated from the International Committee on Taxonomy of Viruses (ICTV) website (https://ictv.global/taxonomy).

Although the complete picture of marine RNA viruses is still emerging, minimal efforts have been made to outline the ecology and significance of marine RNA viruses in their specific niches due to biases in sampling and culturing techniques. Accordingly, it is becoming increasingly clear that our knowledge of marine RNA viruses needs to be considered beyond their clinical and economical effects and in the global context. This review examines our current knowledge of marine RdRp‐encoding RNA viruses (kingdom: Orthornavirae), particularly since the advent of next‐generation sequencing (NGS) methods, and highlights their basic features and a novel taxonomic system suitable for the era of meta‐omics (Figure 1). Finally, we further discuss the theoretical and computational approaches for revealing the marine RNA virus world as well as future challenges and prospects from various perspectives.

## CHARACTERISTICS OF MARINE RNA VIRUSES

### Biological characteristics

Marine RNA viruses have complex genomic features and morphological characteristics. The genome carries the biological information of an organism, and the morphological structure of viral particles is important for the study of the infection mechanism of viruses.

RNA viruses use RNA as genetic material. Their genomes include diverse, complex structures, such as mixes of segmented, unsegmented, and circular genomes. Traditionally, RNA viruses can be divided into single‐stranded RNA (ssRNA) viruses and double‐stranded RNA (dsRNA) viruses. One of the major differences between RNA viruses is their gene expression pattern: positive‐sense ssRNA viruses are translated directly, and negative‐sense ssRNA viruses are translated after conversion to positive‐sense RNA, and the dsRNA may transcribe positive‐sense RNA, which can be used as messenger RNA and replicated to create a new dsRNA viral genome [34]. The ancient gene, RdRp, is the only sequence domain conserved in all RNA viruses [35] and is considered the ancestor of several polymerases and one of the first genes in the peptide‐RNA world, widely used to study the origin of viruses [36, 37]. RNA viral genomes are typically 4–12 kb in size, and the largest known RNA viral genome is that of coronaviruses (41 kb). The main factor limiting RNA viral genome size is low replication fidelity, with the RdRp introducing approximately 10^−4^ errors per nucleotide [38, 39]. This property of RdRp allows RNA viruses to produce different descendants with different genotypes in a short generation time [40]. The high mutation rate leads to overlapping genes in the genomes of RNA viruses, thus leading to genome compression [41]. The biochemical basis for the low replication fidelity of RNA viruses is the lack of proofreading, repair, and postreplication error correction mechanisms during replication, as well as the lack of a replicase system, which makes RNA viruses unstable and prone to mutation [42, 43]. They often show frequent virus–host recombination, horizontal gene transfer, gene gain or loss, and complex rearrangements, which greatly complicate their genetic diversity. This instability makes RNA viruses more susceptible to mutation and, at the same time, makes RNA viruses more unfavorable to study, either by isolation and culture or by metagenomic approaches [44].

RNA viruses in different families vary in their morphology, such as capsid diameter and tail length. Methodologically, variance in virus capsid diameter and tail length can be observed by quantitative transmission electron microscopy [45]. The high degree of order (up to the Å scale) in the secondary/tertiary/quaternary structure of viral capsid proteins constitutes the different morphologies of the viral capsid—spherical/icosahedral and cylindrical/helical [46]. The spontaneous assembly process of the external capsid of RNA viruses is driven by electrostatic interactions between the positive charge on the capsid protein and the negative charge on the genome [47]. Encapsulation of RNA by artificially designed viral capsid proteins could be a new means of protecting and delivering RNA information [48]. The average capsid size of nontailed viruses in the ocean is 54 nm, and according to the nontailed virus cortovirus PM2 currently found in the ocean, they have capsid‐associated lipids [49]. As previously reported, nontailed viruses are dominant (79% of all viruses on average) in surface water, and RNA viruses may account for 16%–100% of the nontailed viruses observed [45, 50]. Because of the difficulty in isolating and culturing RNA viruses from the marine environment, more research is needed for RNA virus morphology.

### Hosts of marine RNA viruses

Viruses cannot exist without a host, and they may be present in every cellular life form [51]. Marine RNA viruses have been found in both eukaryotes and prokaryotes. Upon infection of the host, the virus either directly lyses cells and multiplies or forms RNA phages (only in prokaryotes), and the multiplication of phages occurs mainly through lysis or lysogenic infection [52, 53]. Lytic infection of RNA virus means that the virus replicates after entering the host cell, leading to lytic infection of the host cell. Lysogeny means that the virus gene is integrated with the host gene and does not produce progeny virus particles, but the virus gene can replicate with the host gene and pass along with the division of the host [54]. There are several ways to identify the host of RNA viruses: One is through experimental observation, and the other is through reasonable prediction from the data set. The following methods are commonly used for metagenomic host prediction: (1) detecting whether the host and virus taxa contain consistent clustered regularly interspaced short palindromic repeat (CRISPR) spacer sequences; (2) determining the host based on the host of sequences encoding tRNAs and AMGs in the viral genome; (3) determining the host based on the similarity between viral genomic fragments and potential host gene fragments; (4) predicting hosts based on the abundance and expression levels of viruses and potential hosts in the samples; (5) predicting hosts based on similar tetranucleotide frequencies between viruses and potential hosts [24, 55, 56]. RNA viruses interact with their hosts. RNA viruses bind to antiviral competent restriction factors in the host during their life cycle, inhibiting viral replication and expression [57]. Conversely, RNA viruses induce the production of proteins that degrade restriction factors to inhibit host gene expression during the life cycle of the infected host [58]. RNA viruses also induce host expression of proviral replication RNAs, such as lncRNA‐ACOD1 [59]. RNA virus–host interactions play a vital role in the understanding of virus evolution and ecological changes. The presence of shared RNA viruses in hosts such as protozoa, animals, and plants explains horizontal viral transfer between different hosts as a central aspect of RNA virus evolution [44]. It has been suggested that the cosegregation of RNA viruses with their hosts provides the basis for cross‐species transmission and explains the macroevolution of RNA viruses [60]. Virus–host interactions can affect ecosystems, disease transmission, and other areas relevant to human life. For instance, the interactions between viruses and their hosts can control prokaryotic mortality, release inert organic matter, and compensate for suppressed host metabolic pathways in deep‐sea ecosystems [2, 61, 62, 63]. And at deep‐sea hydrothermal vents, RNA viruses can lyse host cells to control the size of host populations and affect host community structure by killing specific microorganisms. However, viruses also affect the host by manipulating bacterial metabolism to compensate for suppressed host metabolic pathways, thus helping the host to adapt to extreme environments [64].

### Abundance of RNA viruses in the marine ecosystem

Viruses are an integral part of ecosystems and are found in environments, such as soil, humans, and oceans. Just as RNA viruses infect human health, marine RNA viruses affect ocean health and the planet's well‐being [65]. Unlike other environments, the ocean is regarded as the origin of life, and RNA viruses are capable of rapid gene transfer in flowing seawater. Deciphering marine viruses' role, behavior, and function of marine viruses is “one of the great mysteries of this century” [66]. Temperature, ocean currents, host distribution, and biogeography can all impact host abundance and virus abundance [67, 68]. In the marine environment, RNA viruses from low‐ to high‐productivity systems ranged from 10^8^ virus particles per liter to over 10^11^ virus particles per liter [69], and the distribution of RNA viruses is influenced by sea latitude. For example, Taraviricota has a high abundance in temperate and tropical zones, and the ‐ssRNA phylum “Arctiviricota” has the highest abundance in Atlantic Arctic waters [26]. Abundant RNA viruses in the ocean have reshaped our understanding of the early evolution of viruses. Moreover, researchers have identified AMGs in marine RNA viruses that have important effects on marine carbon export [20]. Marine biofouling causes damage to biodiversity and marine ecosystems, through which RNA viruses can be transferred to the global ocean. Spreads of abundant RNA viruses introduce diseases, affect human marine activities, and compromise sustainable human development [70]. With the expansion of RNA virus studies, many problems emerged, such as host sampling for many RNA viruses is insufficient [26], and there are limitations to environmental RNA virus abundance measurements. Solving these issues will require more virus and sample data.

### Adaptation of viruses in the marine environment

Extreme marine environments, such as deep‐sea, polar, and hot regions, hydrothermal vents, and areas of high pressure or salinity, are close to the limits of life [71]. Virus communities adapt to extreme environments by skewing the coding frequencies of specific amino acid residues [72, 73]. Extreme environments do not increase the mutation rate of viruses, but selection pressure from the local environment can increase the abundance of certain types of viruses [74]. The good environmental adaptability of RNA viruses in extreme environments is associated with their high mutation rate. The high adaptive capacity of RNA viruses facilitates the generation of populations consisting of mutant profiles with broad phenotypic and genetic heterogeneity [75, 76]. In these populations, mutant variants that are dominant under selection pressure can be retained as minority variants and rapidly selected when the virus is again exposed to the same selection pressure. Such properties make RNA viruses more adaptable to fluctuating selection pressures [76, 77]. The unique ecological stresses of extreme marine environments may allow viruses and their microbial host communities to synthesize new compounds with different biological activities, and these communities play an important role in biogeochemical cycles [71].

## DEEP MARINE VIRUS DISCOVERY CONTRIBUTES TO THE MEGATAXONOMY OF RNA VIRUSES

Substantial efforts have been made to understand pathogenic viruses that infect humans, economically or socially important animals, and plants [78, 79, 80], and the development of NGS at a spectacularly rapid pace has led to a new age of virus discovery and taxonomy [81]. It is widely recognized that viral information is extremely abundant in the biosphere and public databases worldwide, far beyond what virologists have captured in laboratory studies [82]. Accordingly, viral taxonomy, including the practice and science of virus hierarchical classification, has changed dynamically over the last two decades, during which RNA viruses have received frequent and distinct taxonomic updates (Table 1).

**Table 1:** History and updates of the megataxonomy of RNA viruses

	In five‐rank hierarchy	In 15‐rank hierarchy		
		Five phyla		Proposed 10 phyla
Criteria for	Virus biology			
classification	Epidemiology	Phenotypes according to genome and metagenome data		
	Host factors	Genetic relationship inferred from sequence similarity		
	Sequence relationship from genome analysis	Biological data are not essential		

**Top rank of RNA viruses**	**Order**	**Phylum**	**Phylum**	
Number of species in Orthornavirae	1825 (2016)^a^	4060 (2021)^b^	More than 5000 new species discovered	
			Duplornaviricota	
Assigned or	None	Duplornaviricota	Kitrinoviricota	
proposed		Kitrinoviricota	Lenarviricota	
phylum		Lenarviricota	Negarnaviricota	
		Negarnaviricota	Pisuviricota	
		Pisuviricota	Arctiviricota	
			Paraxenoviricota	
			Pomiviricota	
			Taraviricota	
			Wamoviricota	

^a^ Derived from International Committee on Taxonomy of Viruses (ICTV) Master Species List 2016 v1.3, https://talk.ictvonline.org/files/master-species-lists/m/msl/6776.

^b^ Derived from ICTV Master Species List 2021.v1, https://talk.ictvonline.org/files/master-species-lists/m/msl/13425.

### Early five‐rank hierarchy of viruses

Global viruses replicate and express their genomes by several different strategies, which were used by Baltimore in 1971 to classify all known viruses into six groups and later used to introduce a seventh group [83]. The Baltimore framework, based on forms, polarity, and expression of the viruses' nucleic acids, classifies RNA viruses as dsRNA viruses, positive‐sense RNA [(+)RNA] viruses, negative‐sense RNA [(−)RNA] viruses, and RNA reverse‐transcribing viruses. Since this system provides insights into both the replication and transcription of viruses, it is still widely used [84]. However, recent evidence illustrates that viruses in the same Baltimore class do not always share a common ancestor [85], indicating that the system does not necessarily reflect the actual evolutionary relationships between and among different groups.

The earliest research on viruses emphasized molecular virology after laboratory isolation and culture, and the ratification of a new species by the International Committee on Taxonomy of Viruses (ICTV) during the 1970s–1990s was mainly dependent on biological characteristics, such as their properties in vitro, virion structure, and antigenic relationships [86]. Meanwhile, many host factors (e.g., virulence, pathogenicity, host range, and epidemiology) were also considered. On that basis, the ICTV then classified viruses into two different ranks and expanded the virus taxonomic classification system to include a five‐rank hierarchical structure of species, genus, subfamily, family, and order [84]. This hierarchy was in place until 2017, at which time nine orders, 122 families (37 subfamilies), 735 genera, and 4404 species were listed (ICTV Master Species List 2016 v1.3, https://talk.ictvonline.org/files/master-species-lists/m/msl/6776).

### Fifteen hierarchical ranks and a five‐branch hierarchy of riboviria

With advances in Sanger sequencing techniques and the understanding of virus genetics, viruses can be sequenced for their nucleic acid, even before their biological attributes are characterized [87, 88]. Accordingly, inferences from virus sequences via divergence estimation and phylogeny construction were combined with experimental and epidemiological factors to assign viruses to taxonomic groups [89, 90, 91]. *Heterosigma akashiwo* RNA virus (HaRNAV), for example, was the first marine RNA virus isolated in coastal British Columbia and was characterized to infect the toxic red‐tide‐forming photosynthetic alga raphidophyte *H. akashiwo*. HaRNAV was the first classified member of the newly named family Marnaviridae in the order Picornavirales according to its host, morphology, virion size, genome size, distinct domain organization, and phylogenetic relationship with viruses in related families [92, 93].

Given that viruses lack common hallmark genes that can be used to construct a unified phylogenetic tree encompassing all viruses, the taxonomy of viruses differs to some degree from that of cellular organisms, particularly for viruses at different higher ranks and in different Baltimore classes. For RNA viruses, the presence of homologous RdRp enables inference of sequence distances and evolutionary relationships between families, which may provide support for novel ranks [84].

In the era of metagenomics, potential viruses in environments or living organisms that show limited similarity with known viruses or cannot be classified outnumber those recognized by the ICTV. A study profiling the transcriptome of marine and inland invertebrate species identified 1445 RNA viruses, some of which were significantly divergent from known families [35]. Despite the fact that contigs derived from mixed virus populations are at risk of artificial chimeras and cannot recover bipartite or multipartite genomes (often seen in RNA viruses), advanced computational and experimental methods (e.g., generating longer reads) increase the potential to resolve these problems [94, 95, 96]. This is why a workshop of the ICTV Executive Committee was held, with experts invited to discuss whether and how viruses discovered by metagenomics can be absorbed into the ICTV taxonomy. As a consequence, a consensus was reached that the proposed taxonomy can be applied to virus genome and metagenome sequence data. Alternatively, phenotypes predicted from genome analysis and distances inferred from sequence comparisons function as fundamental elements for virus taxonomy, while biological data are no longer essential [86].

A 15‐rank hierarchy, placing the global virosphere into eight primary ranks and seven secondary ranks, was discussed and approved by the ICTV Working Group in 2016 as the best equivalent of the complete Linnaean taxonomic system and a novel system for virus taxonomy [84]. The primary ranks include four already existing (order, family, genus, and species) and four newly incorporated ranks (realm, kingdom, phylum, and class), while the secondary ranks include the previously used subfamily rank and six new ranks derived from the primary ranks (subrealm, subphylum, subkingdom, subclass, suborder, and subgenus). This updated taxonomic system serves as a dynamic framework for approving virus taxonomic assignment as virus discovery continues and is inclusive of numerous viruses that remained outside of the former virus taxonomic system and thus reflected biases toward some sampling environments and organisms as well as different virus lineages.

The only realm established later, Riboviria, included RdRp‐encoding RNA viruses from all three Baltimore groups (https://talk.ictvonline.org/taxonomy/p/taxonomy-history?taxnode_id=202107095). A comparative genomic study carried out to reinvestigate the evolutionary relationships within RNA viruses through deep phylogenetic analysis and sequence similarity networks of virus hallmark genes supported the RNA virus RdRp phylogeny containing five major branches [30], which were proposed as phyla Lenarviricota (branch 1), Pisuviricota (branch 2), Kitrinoviricota (branch 3), Duplornaviricota (branch 4), and Negarnaviricota (already established, branch 5), respectively, in the proposed kingdom Orthornavirae (RdRp‐encoding RNA viruses contrasting with RT‐encoding RNA viruses). Though important issues of extreme divergence of RdRp sequences and subjectivity in the process of manual curation have received intense discussion, several complementary methods like using RdRp primary sequence, HMM models, and three‐dimensional (3D) structure supported the practicability of RdRp‐based sequence analysis with some refinement and supplements [10, 26, 31]. The creation of the Riboviria realm and subsequent 5‐branch taxonomic partitioning of viruses was considered the first step in applying the newly proposed rank structure of virus taxonomy and further helped to clarify an obvious relationship between RNA viruses, which facilitated the discovery, classification, and nomenclature of emerging RNA viruses [85].

### Ten proposed phyla of global RNA viruses

More large‐scale endeavors have been made to further uncover the marine virosphere. In an up‐to‐date attempt to expand RNA virus diversity in global oceans, Ahmed A. Zayed and colleagues recovered over 44,000 RdRp‐coding contigs and 6686 complete or near‐complete RdRps via an optimized iterative search approach from 771 Tara Ocean metatranscriptomic resources globally collected from the world's five oceans and 143 new metatranscriptomic data points from the Arctic Ocean [26]. More importantly, this groundbreaking and highly representative work hinted that six newly established “megaclusters” may correspond to five new phyla (named “Arctiviricota,” “Paraxenoviricota,” “Pomiviricota,” “Taraviricota,” and “Wamoviricota”), doubling the number of known phyla in the kingdom Orthornavirae.

Additionally, revision of the origin and early relationships of reported orthornaviran phyla was considered based on analysis at the phylogenetic, structural, and genomic levels, thus complementing the primary sequence‐inferred phylogeny and representing a relatively robust roadmap. These analyses suggested polyphyly of viruses in the phylum Duplornaviricota (dsRNA viruses), while a previous study indicated that these dsRNA viruses evolved from (+)RNA viruses, with the former possibility supported by more evidence and revealing three different phyla. Meanwhile, the newly proposed phylum “Taraviricota,” placed in a phylogenetic position linking retroelements and orthornavirans, was considered a potential early evolutionary origin of other orthornaviran phyla.

Although challenges and biases toward sample types remain, continuous methodological and intellectual advances, especially in deep metagenomics and evolutionary virology, shed light on the discovery and taxonomy of global viruses and reveal the secrets of the marine RNA virosphere.

## META‐OMICS OF VIRUS DISCOVERY AND ANNOTATION

### Before the modern metagenomic era

The earliest report on a marine RNA virus was initially based on the isolation of RNA viruses infecting marine animals economically important for aquaculture [97]. Since then, several protistan RNA viruses in the sea have been discovered and found to have a wide host range [98, 99]. Although instructive and informative, these virological studies predominantly focused on molecular characteristics [100, 101, 102], indicating that an understanding of the ecology of marine RNA viruses remains to be reached, including their diversity, biogeography, interactions with hosts, evolutionary status, and environmental contributions.

Environmental RNA virus discovery by isolation‐independent approaches subsequently arose and revealed the diversity and crypticity of the RNA viral community in the sea [103, 104, 105]. For viral genomes, unlike the genomes of cellular organisms, no universal and conserved genes are shared by all seven Baltimore classes of viruses. However, polymerase chain reaction (PCR) targeting broadly conserved hallmark genes has been applied to amplify and discover diverse groups of viruses. As early as 2003, degenerate primers targeting RdRps combined with reverse transcription‐PCR (RT‐PCR) were used to survey the presence and diversity of picorna‐like viruses in seawater [106]. Four monophyletic clades of picorna‐like viruses probably representing at least two new viral families were identified using sequence analysis, showing the potential of a single‐gene survey for RNA viral discovery in marine environments [107]. In addition to viral discovery, more recent studies based on RdRp‐targeted RT‐PCR also revealed the temporal and spatial variation of picorna‐like ssRNA viruses in the coastal areas of British Columbia by combining location and time‐series sampling [108], the predominant role of picornavirads in the pool of RNA viruses in tropical coastal seawater samples through additional metagenomic methods [109], and the impact of RNA viruses on the community composition of the potential host with extra amplicon sequencing of marker genes [108, 110]. Notably, these data gave an initial glimpse into the diversity, richness, and ecology of a particular RNA virus group in the ocean, but this method provided little information about genetic diversity (i.e., other viral genes) and is inapplicable to the global and comprehensive assessment of RNA viruses, especially at the genome and function levels, because of underrepresentation of all viral groups and the lack of complete genetic materials.

### Marine RNA viromics based on viral particle enrichment

The limitations of the culture‐dependent approach and single‐gene probing motivated the advent of modern metagenomics, a concept first defined as the study of collective genetic material cloned from natural environmental samples [111] and subsequently expanded to the direct study of microbial communities without laboratory isolation and cultivation of individual species [112]. Viromic studies of inchoate marine viruses characterized by underestimated sampling sites and with only double‐stranded DNA viruses revealed [113, 114] and necessitated large‐scale sampling and sequencing efforts such as the Sargasso Sea project [103] to provide raw data and insights into the community dynamics of marine viruses [99, 112].

The first RNA viral community‐focused study investigated the community structure of uncultivated RNA viruses in four aquatic environments by constructing random reversed‐transcribed whole‐genome shotgun libraries and shed light on the persistent and widespread existence of RNA virus assemblages (including viruses in the orders Picornavirales, Mulpavirales, and Tolivirales and other unclassified and unknown viruses) with relatively short genomes in the sea [104]. This study verified the feasibility of RNA viral metagenomics as a practicable approach for unveiling the hidden marine RNA virosphere. Compared with traditional methods, marine viromics has shown superiority in recovering viral contigs bypassing complex isolation and culturing procedures, thus the number of documented uncultivated viruses has exceeded isolated viruses as early as 2016 [28]. Meanwhile, marine virus discovery through meta‐omics is competent to avoid sampling biases and provide global landscape and ecological perspectives [20, 26, 115]. In the years that followed, an increasing number of projects were carried out to explore viruses in different environments, such as tropical seawaters [109, 116], Atlantic coastal seas [117, 118], the southern Indian Ocean [119], the Canadian Arctic [120], the Baltic Sea [121], and the deepest trenches [68], and from marine organisms, such as diatoms [122], oomycetes, marine arthropods, and eukaryotic phytoplankton [27]. Likewise, RNA viral metagenomics contributed greatly to expanding our understanding of RNA ecological characteristics, including the abundance and diversity [7], temporal dynamics [118], biogeographic distribution [120], and host interactions [120] of marine RNA viruses.

**Figure 2:**
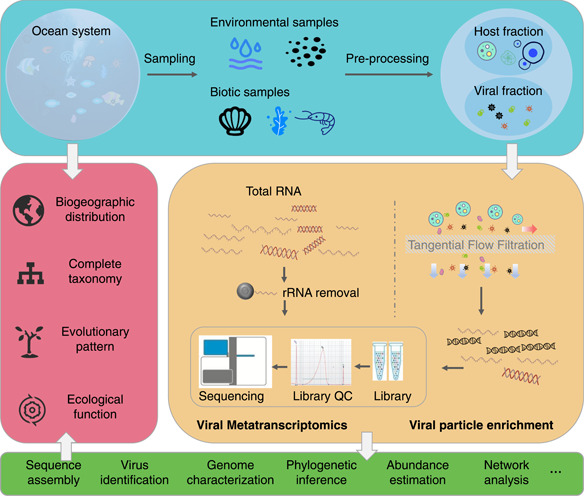
Summary of a marine viral meta‐omic pipeline. Step (1): experimental design and protocol. This step is often placed little importance in the meta‐omics study but could provide substantial information if carried out carefully. Step (2): different approaches to capturing viral nucleic acid provide discrepant information about marine RNA viruses and their hosts. Step (3): various and personalized analyses after assembling virus genomes. Step (4): detailed explanation in the context of viruses, hosts, and environment and inference from a global view.

RNA viromics contributed significantly to viral discoveries for many research priorities, consisting of capturing viral genetic materials directly without the need for isolation and cultivation, removing background DNA, and providing a metastable approach with no wet laboratory necessary. Operationally, a representative particle‐based RNA viromic workflow entails the following steps: sampling and cellular fraction filtration, viral fraction concentration, nucleic acid extraction and amplification, library preparation and high‐throughput sequencing, and finally, downstream personalized data analysis [24, 25, 123, 124]. Among these main procedures, several important biases exist, indicating that careful consideration must be made to avoid misinterpretation. A viral concentration and purification process are generally performed to reduce the sample volume and facilitate subsequent steps, with tangential flow filtration (TFF), iron chloride flocculation, ultracentrifugation, and CsCl gradient centrifugation frequently used [125]. Through different cutoffs [126, 127], these methods maintain the integrity of viruses but introduce bias in the virus types, as they present diverse viability and efficiency levels for different types of viruses [128, 129]. Exogenous nucleic acid contamination, probably derived from samples, reagents [85], and sequencing platforms [130], should be safely controlled and minimized to guarantee the accuracy of the result via DNA/RNA nuclease treatment, experimental control design, and cautious consideration of viruses with low abundance (Figure 2).

### Marine viral metatranscriptomics

Various factors, including accumulating RNA sequences in databases, better experimental protocols, and more sensitive in silico tools, brought about the application of metatranscriptomics—the total transcriptome in a given niche—to RNA virus discoveries over the past decade, which is superior in offering information about RNA viruses (with RNA‐based genomes) as well as the transcriptome of organisms with a DNA‐based genome (i.e., cellular lifeforms and DNA viruses) [60]. A typical RNA viromics protocol includes sampling (from either environments or organismal tissues), cell lysis, DNA removal, and RNA extraction and concentration (via mRNA isolation or rRNA removal), followed by reverse transcription, sequencing, and data analysis (Figure 2) [44, 131, 132].

By virtue of being unbiased, widely available, and cost‐efficient, metatranscriptomics has helped uncover cryptic and diverse RNA viruses among a wide range of sample habitats, including environmental samples [133, 134], microorganisms [135], plants [136], invertebrates [17, 78], and vertebrates [137]. These studies made predominant contributions to the knowledge of the hidden diversity [17], evolutionary scenarios [44, 131], genetic and phylogenetic diversity, and biogeographic distribution of marine RNA viruses. Furthermore, as a multifaceted tool providing information from both viruses and their hosts, this technique has been used to detect active viral infections and infer virus–host relationships [138] and interactions [1] in situ.

Along with the accumulation of viral metadata in public databases, several advanced bioinformatic tools based on different principles [139] can detect and classify RNA viruses after identifying proteins from recovered metagenomic or metatranscriptomic contigs, including similarity‐based Basic Local Alignment Search Tool searches [140], the k‐mer approach in VirFinder [141] and Kraken2 [142], and Vibrant [143] and VirSorter2 [144], which use domain abundance and/or key homologous genes. These approaches have been benchmarked for their performance in discovering viruses in terms of precision, recall, and F1 scores across simulation conditions [139]. Both contigs length and taxonomic complexity can influence the average performance of these tools and researchers can select the appropriate tool according to their own needs. Notably, correction of possible errors is usually achieved by laboratory experiments (RT‐PCR based on designed primers [11, 35, 115]) and computational confirmations (considering authenticity in the context reference RdRps from known RNA viruses [115, 131], or mapping reads into viral contigs and estimating abundance [11, 14]). Simultaneously, deep phylogenetic analysis centered on RdRp proteins appears to be informative and practicable in inferring the evolution and taxonomy of marine RNA viruses [10], although it does face challenges in cases of extreme sequence divergence and is robust only in the case of distinct primary sequence similarity [31]. Notably, it is suggested that conclusions drawn from RdRp‐based phylogenies be carefully considered in the context of other evidence, such as RdRp 3D structure and clustering, existence and permutation of other domains, and whole‐genome features. Additionally, the ecological roles and biogeographic patterns of marine RNA viruses can be explored when sufficient niche background information is available.

### Future challenges and perspectives

NGS and viromics have revolutionized virus discovery and led to an explosion of known and unknown viral sequences, which, to a certain degree, complicates an accurate understanding of the actual marine RNA virosphere. Unlike defining prokaryotic DNA virus–host relationships, it is usually not adaptable to detecting eukaryotic RNA virus hosts via approaches based on CRISPR spacer matches or sequence similarity [145]. Even when sampling tissues directly, the inferred species cannot be verified as the host since the viruses may actually infect internal microbes or a dietary component. Studies aiming at exploring promising mechanisms (like infection‐induced sequence features and coexistence relationships) or new technical innovations are encouraged to define virus–host associations and to improve accuracy and applicability. Second, improvement of viral genome completeness is urgent, necessitating greater sequencing depths, advances in long‐read sequencing, and better assembler tools. Another challenge is that few bioinformatic software programs can be utilized to precisely classify RNA viruses showing little or no similarity with known viruses [146]. Last, large technical gaps remain in the isolation of RNA viral particles from environmental and poorly studied biological samples, as well as in viral culturomics and molecular verification. Progresses in this field will contribute to a deeper understanding of RNA virus biological features and broader application of RNA viruses by human beings at the community and/or ecosystem levels.

Virus discovery through meta‐omic sequencing may represent a field without an obvious trajectory and help illuminate underexplored RNA viral “dark matter,” representing unknown aspects regarding marine RNA viruses, like, taxonomic, metabolic, and functional diversity. The evolutionary processes of different types of viruses and the broad taxonomy of the virus world are expected to be refined through large‐scale and unbiased viromics. In addition, as previous ecological function studies focused mainly on DNA phages, there has been less evidence for the roles of RNA viruses. Considering their abundance and host range, marine RNA viruses have been reported to be associated with the modulation of host diversity [121], algal bloom [138], host metabolic reprogramming, and ocean carbon export [20]. So, it is of utmost importance to clarify what impacts marine virosphere and what marine viruses impact. Particularly, the roles of marine RNA viral groups involved in carbon, nitrogen, sulfur, and other earth element cycles can be determined at the genomic and metabolic levels, by integrating the metabolite spectrum and geographical‐climatic characteristics, thus providing a theoretical basis for the development of marine viral resources and the use of RNA viruses to determine and regulate land‐ocean‐atmosphere carbon fluxes. More studies are still needed to support a more complete functional characterization of these RNA viruses.

## CONCLUSION

In this review, we highlight that the global search for RNA virosphere components via meta‐omics has helped to unravel cryptic and diverse RNA viral populations in marine environments and shed light on their biogeographic distribution and evolutionary patterns. However, these findings also hint at the situation where gaps still exist, particularly in the current virus hierarchical taxonomic system in the era of information explosion and functional annotation in this specific ecosystem. Additionally, more advanced sequencing (generation of longer reads) and computational methods (assembly of viral genomes as completely as possible) are needed to further improve our understanding of marine RNA viruses both already known and yet to be discovered. With a large number of RNA viruses and their relationships with hosts and environmental elements revealed by using multifaceted approaches, the potential roles that marine RNA viruses play, like shaping carbon cycling in soil, might emerge and thus redefine their ecological status in nature.

## AUTHOR CONTRIBUTIONS

Meng‐en Liao and Jie Cui conceived the idea. Meng‐en Liao, Mang Shi, and Jie Cui discussed the content. Meng‐en Liao, Yunyi Xie, and Jie Cui wrote the first draft of the manuscript. Jie Cui contributed to critical revision.

## CONFLICT OF INTEREST

The authors declare no conflict of interest.

## Data Availability

This manuscript does not generate any script and data. All authors read and approved the final manuscript. Supplementary materials (figures, tables, scripts, graphical abstract, slides, videos, Chinese translated version, and updated materials) may be found in the online DOI or iMeta Science http://www.imeta.science/.
